# Reparameterizable large kernel attention networks for infrared image super-resolution

**DOI:** 10.1038/s41598-025-24193-3

**Published:** 2025-11-18

**Authors:** Ran Wei, Linze Zuo, Xuesong Wang, Xianyu Wu

**Affiliations:** 1 Department of Rehabilitation Engineering, China Civil Affairs University, 102600 Beijing, China; 2 College of Mechanical Engineering and Automation, Fuzhou University, 350108 Fuzhou, China

**Keywords:** Super-resolution reconstruction, Infrared image, Neural network processor, Convolutional neural network, Computer science, Electrical and electronic engineering

## Abstract

To address the challenge of balancing reconstruction performance and inference speed in the existing infrared image super-resolution algorithms, this paper introduces a novel Large Kernel Reparameterization Attention mechanism. Based on this, we propose the reparameterizable large kernel attention network for infrared image super-resolution. During training, a multi-branch large kernel network is employed to fully extract information, while at inference time, it is equivalently transformed into a single-branch large kernel network, achieving a trade-off between processing performance and inference speed. Compared to state-of-the-art methods, our approach improves the average PSNR on a self-constructed infrared dataset by 0.0008 dB. Additionally, on the RK3588 Neural Processing Unit, it requires only 37ms to perform 4$\times$ super-resolution on 320$\times$180 images.

## Introduction

Infrared imaging technology allows us to observe parts of the spectrum beyond visible light, which enables the conversion of invisible infrared radiation into visible images, expanding our perceptual range. However, infrared images obtained by detecting the heat radiated by objects often suffer from low resolution, insufficient contrast, and blurriness, posing challenges for research and applications. The low resolution affects clarity and detail representation, limiting their usefulness.

Improving imaging quality through hardware enhancements is costly in terms of industrial expenses and effort, and the ultimate performance improvements are constrained by insurmountable physical limitations. In contrast, infrared image super-resolution (SR) reconstruction technology offers a cost-effective solution by recovering high-resolution (HR) infrared images from low-resolution (LR) counterparts. This approach meets the practical demand for high-definition infrared images, opening up new possibilities for the application and dissemination of infrared imaging technology across various fields.

Infrared image super-resolution typically employs general image SR methods. Zhang et al.^1^ were the first to use compressed sensing for SR image reconstruction. They downsampled the SR and high-resolution images to capture high-frequency noise information, which was then fed into Convolutional Neural Networks (CNNs) to learn nonlinear mappings. Experiments demonstrated that using CNNs to represent nonlinear mappings could enhance texture information in reconstructed infrared images. Kwasniewska et al.^2^ developed a wide receptive field residual network using dense connections, confirming that a wide receptive field effectively enhances low-contrast infrared images. Yuan et al.^3^ proposed a gradient residual attention network based on CNNs, utilizing gradient operators to better extract features from infrared images. Zou et al.^4^ constructed an infrared image SR model similar to U-Net using residual networks, incorporating multi-receptive field modules to extract high-frequency and low-frequency features and achieving commendable results. Recent research has further explored the integration of physical models and deep learning: Zhang et al.^5,6^ significantly improved the spectral fidelity of image reconstruction by combining image degradation models with deep priors, offering new references for image restoration.

Although these models successfully reconstruct high-quality infrared images, they heavily rely on large datasets. In some fields, constructing datasets is challenging due to expensive equipment or a limited natural environment. While self-supervised learning^7^ and semi-supervised frameworks^8^ boost model robustness in data-scarce conditions, transferring knowledge from large pre-trained datasets yields superior reconstruction performance in target domains. Consequently, transfer learning has emerged in many methods across various image processing domains^9–11^.

In the field of infrared image SR, Almasri et al.^12^ used residual blocks to separately extract information from visible and infrared images, followed by fusion. Their experiments demonstrated that visible light images help improve the high-frequency details of infrared images. Huang et al.^13^ proposed PSRGAN (Progressive Super-Resolution Generative Adversarial Network), which leverages visible images and 100 pairs of infrared images to enhance the restoration performance of infrared images. The proposed PSRGAN achieved excellent infrared SR performance by fine-tuning a pretrained network using only 55 infrared images.

In practical applications, super-resolution algorithms often need to be deployed on resource-constrained devices. Due to their high computational complexity, existing algorithms struggle to balance performance and speed on such devices^14,15^. In recent years, the model reparameterization technique has emerged as an effective network optimization strategy, converting complex modules of trained models into simplified structures, significantly improving the deployment capability of models in resource-constrained hardware environments. In the field of computer vision, the ACB^16^ (Asymmetric Convolution Block) technique integrates asymmetric convolution structures with standard convolution substrates, effectively enhancing the performance of convolutional neural networks. On the other hand, the RepVGG architecture achieves a dual breakthrough in accuracy and speed in image classification tasks through a stacking design of multi-layer reconstructed convolutions^17^. With innovation based on the reparameterization technique, lightweight super-resolution models have made significant progress. The FIMDN framework, proposed by AIM2020, enhances the IMDN network with ACB, verifying the feasibility of improving super-resolution performance while maintaining inference efficiency^18^. In terms of feature extraction, DBB (Diverse Branch Block) adopts a multi-branch structure similar to Inception, effectively capturing diverse features during the training process. This innovation has been widely applied to various network architectures^19^. Inspired by RepVGG and DBB, the Edge-oriented Convolution Block (ECB) targeting mobile devices achieves a balance between computational efficiency and visual quality through a designed reconstruction convolution method for real-time applications^20^. However, these reparameterization techniques still face challenges in the application of deep networks, mainly due to the high training complexity involved and limitations imposed by the local receptive field.

To address the challenge of achieving a balance between performance and inference speed for infrared image SR algorithms on resource-constrained platforms, this paper proposes a large kernel reparameterization attention mechanism. Based on this, we introduce the reparameterizable large kernel attention network for infrared image super-resolution (REPLKASR) network. Inspired by RepVGG, the method proposed in this paper applies the large kernel convolution attention module to the reparameterization method. By employing a multi-branch large kernel network during training to fully extract infrared image features and equivalently transforming it into a single-branch large kernel network during inference, we achieve a trade-off between speed and performance. Unlike existing reparameterization techniques focusing on small kernel optimization (e.g., the 3$\times$3 convolution kernel in ECB/RepVGG), our method achieves fundamental expansion of the receptive field by fusing 5$\times$5 large convolution kernels, while maintaining the deployment efficiency of structural reparameterization. To reduce the dependence of model training on large-scale infrared datasets, this method first pretrains on visible light datasets to obtain basic feature representations, and then fine-tunes on infrared datasets to achieve cross-modal knowledge transfer. On the RK3588 Neural Processing Unit (NPU), our approach can perform 4$\times$ super-resolution on 320$\times$180 images in just 37 ms, meeting the requirements for real-time super-resolution of infrared images.

The main contributions of this paper are threefold:

1. This study innovatively designs a large kernel reparameterization unit (REPLKA), which extends existing reparameterization techniques (e.g., ECB). By expanding the convolutional kernel parameters from the standard 3$\times$3 to 5$\times$5, REPLKA dynamically integrates features using a multi-branch structure during training while converting to a single-branch architecture during inference. This approach not only effectively enhances static and dynamic feature extraction capabilities but also ensures deployment efficiency.
2. This study develops an infrared super-resolution network based on the attention mechanism using large kernel reparameterization. This network integrates the REPLKA module and ECB architecture to optimize infrared image reconstruction performance through multi-scale feature representation. Additionally, the training strategy incorporates knowledge transfer, effectively resolving the data scarcity problem.
3. To validate the feasibility of the proposed method, this study chooses the Rockchip RK3588 development board as the hardware deployment platform and performs comprehensive quantitative and qualitative evaluation of existing state-of-the-art methods on numerous benchmark datasets. The experimental results demonstrate that, in comparison to conventional image super-resolution approaches, REPLKASR attains superior peak signal-to-noise ratio (PSNR) and structural similarity index (SSIM) performance while employing fewer parameters.

The subsequent sections of this paper are structured as follows: Chapter 2 details the design principles of the REPLKA module and the large-kernel convolution reparameterization mechanism, while introducing the overall architecture of the REPLKASR network; Chapter 3 systematically presents the experimental design, including the training strategy, benchmark dataset evaluations, and deployment outcomes on the RK3588 NPU platform; Chapter 4 provides an in-depth discussion of the method’s limitations, ablation study results, and potential future extensions; finally, Chapter 5 concludes with the core contributions.

## Proposed method

### Network architecture

**Fig. 1:**
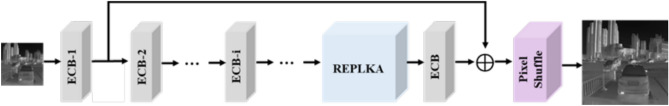
Overview of our RepLKASR.

The structure of the proposed REPLKASR network is illustrated in Fig. 1 and consists of three main components: a shallow feature extraction module, a deep feature extraction module based on cascaded ECB modules and REPLKA modules, and a high-quality image reconstruction module. The ECB and REPLKA modules will be introduced in Sections 2.3 and 2.4, respectively.

The shallow feature extraction module consists of a single ECB module, given the input low-resolution image ${{{I}}_{\text {LR}}}\in {{R}^{\left( 3\times \text {H}\times \text {W} \right) }}$,where H and W represent the height and width of the LR image, respectively. The application of the shallow feature extraction module is represented as $\text {ECB}\left( \cdot \right)$,utilized for extracting shallow features.

The process is represented as follows:

1 $$\begin{aligned} {{{F}}_{\text {p}}}=\text {ECB}({{{I}}_{\text {LR}}}). \end{aligned}$$

Next, the shallow features are passed onto the deep feature extraction module to obtain deeper and more abstract high-level features. This process can be described as follows:

2 $$\begin{aligned} {{{F}}_{\text {r}}}\text {=}{{{f}}_{\text {DF}}}\text {(}{{{F}}_{\text {p}}}\text {)}. \end{aligned}$$

Where ${{F}_{\text {r}}}$ represents the deep feature maps, ${{f}_{\text {DF}}}\left( \cdot \right)$ represents the deep feature extraction module, which includes multiple cascaded ECBs and a REPLKA module. Intermediate features ${{F}_{1}}$, ${{F}_{2}}, \ldots , {{F}_{n}}$ are progressively extracted. The specific process is illustrated as follows:

3 $$\begin{aligned} & {{{F}}_{{i}}}\text {=}{{{f}}_{\text {ECB}{_{\text {i}}}}}\left( {{{F}}_{{i-1}}} \right) \text {, }\!\!~\!\!{ i=1,2,......,}{n}. \end{aligned}$$

4 $$\begin{aligned} & {{{F}}_{{n}}}=\text {REPLKA(}{{{F}}_{{n-1}}}{)}. \end{aligned}$$

Where ${f}_{{\text {ECB}}_{i}}(\cdot )$ represents the i-th ECB, $\text {REPLKA}(\cdot )$ denotes the REPLKA module, and *n* indicates the number of ECBs. Subsequently, ${{F}_{\text {r}}}$ and ${{F}_{\text {p}}}$ are fed into the image reconstruction module to complete the super-resolution reconstruction of the image. This process can be described as:

5 $$\begin{aligned} {{{I}}_{\text {SR}}}\text {=}{{{f}}_{\text {RC}}}\text {(}{{{F}}_{\text {p}}}\text {+}{{{F}}_{\text {r}}}\text {)}. \end{aligned}$$

Where ${{I}_{\text {SR}}}$ represents the reconstructed SR image, ${{f}_{\text {RC}}}$ denotes the upsampling module, which consists of sub-pixel convolutional layers.

### Structural reparameterization

**Fig. 2:**
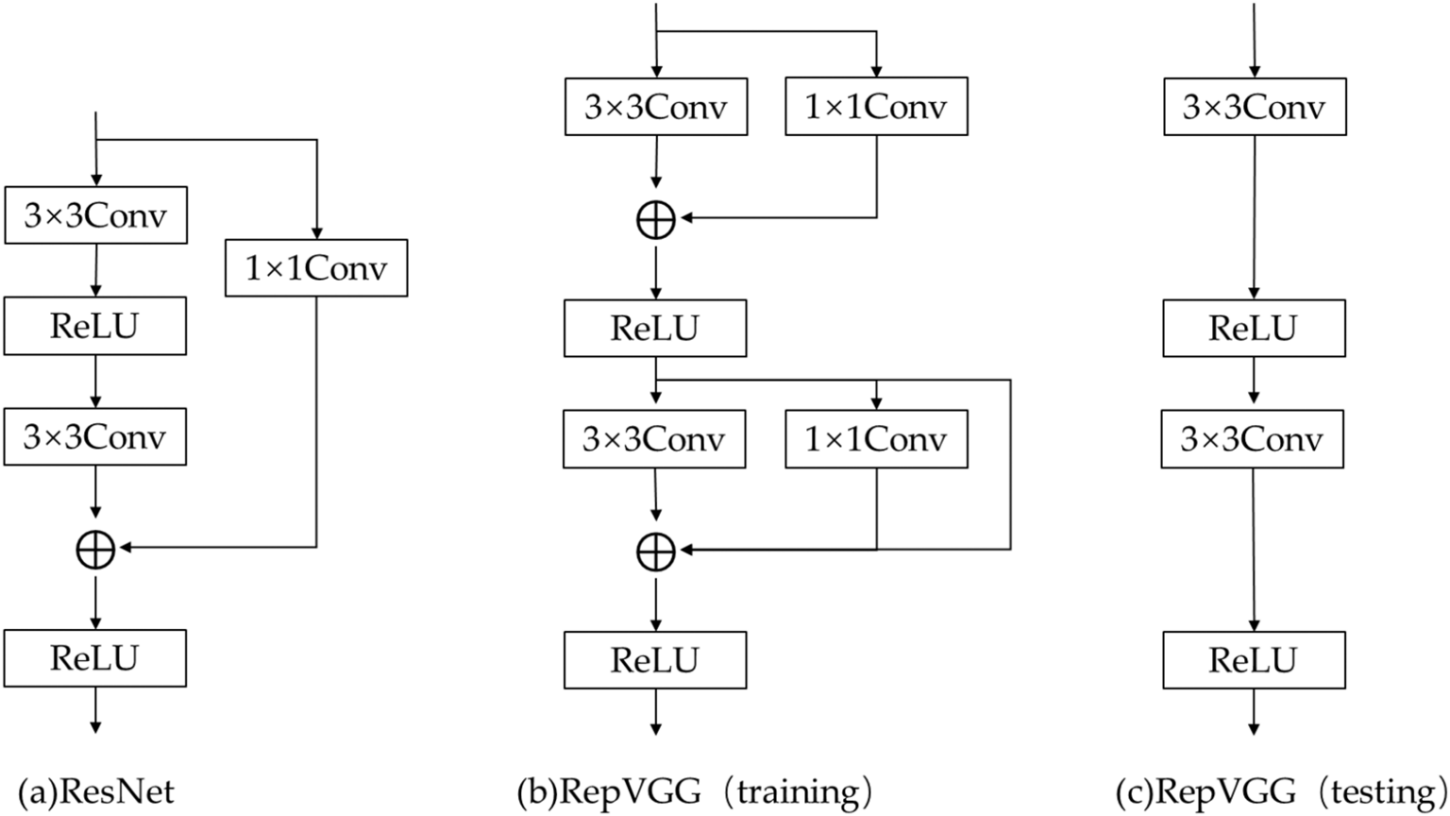
Architecture diagrams of ResNet and RepVGG.(**a**) ResNet; (**b**) RepVGG architecture during training; (**c**) RepVGG architecture during testing.

During training, neural networks often utilize multi-branch models similar to the ResNet^21^ style,as illustrated in Fig. 2(a), where parallel branches generally enhance the model’s representational capacity. Each branch can learn different features and ultimately enhance the model’s performance by combining the features through fusion mechanisms. However, multi-branch network models require multiple memory accesses and writes during inference, resulting in significant time wastage. Additionally, the time consumption increases when multiple branches are merged. By converting the multi-branch network into a single-path model with a VGG-style^22^ architecture during inference, these drawbacks can be overcome.

Figure 2(b) illustrates the multi-branch network structure used during model training, while Fig. 2(c) represents the network structure utilized during inference.

Structural Reparameterization^20^ refers to the process of combining the biases and weights of a pretrained multi-branch network and storing them in a single-branch network model, ensuring consistency between the results obtained from multi-branch and single-branch networks during inference. Structural Reparameterization primarily takes three forms: merging convolutional and batch normalization (BN)^23^ operators, expanding 1$\times$1 convolutional layers into 3$\times$3 convolutional layers, and merging 3$\times$3 convolutional layers on each branch into a single 3$\times$3 convolutional layer.

Since both convolution and BN operators perform linear operations, they can be merged into a single operator. For the BN layer, it mainly includes four parameters: $\mu$ (mean), ${{\sigma }^{2}}$ (variance), $\gamma$ and $\beta$, where $\mu$ and ${{\sigma }^{2}}$ are obtained statistically during the training process, while $\gamma$ and $\beta$ are learned during training. The calculation formula for the i-th channel of the feature map’s BN is shown in Eq. (6), where $\varepsilon$ is a very small constant to prevent the denominator from being zero.

6 $$\begin{aligned} {{{y}}_{{i}}}{=}\frac{{{{x}}_{{i}}}{-}{{\!\!\mu \!\!}_{{i}}}}{\sqrt{{{\!\!\sigma \!\!}^{\text {2}}}{+}\!\!\varepsilon \!\!}}{{\!\!\gamma \!\!}_{{i}}}{+}{{\!\!\beta \!\!}_{{i}}}. \end{aligned}$$

For the feature map *M* of the *i*-th input to the BN layer, it can be represented as Eq. (7):

7 $$\begin{aligned} bn{{(M,\mu ,\sigma ,\gamma ,\beta )}_{:,i,:,:}}=\left( {{M}_{:,i,:,:}}-\frac{{{\gamma }_{i}}}{{{\sigma }_{i}}} \right) +{{\beta }_{i}}. \end{aligned}$$

The weights of the new convolutional layer after the transformation can be calculated using Eq. 8, where *i* represents the *i*-th convolutional kernel, and ${W}'$and ${b}'$ are the new weights and biases.

8 $$\begin{aligned} W_{i,:,:,:}^{\prime }=\frac{{{\gamma }_{i}}}{{{\sigma }_{i}}}{{W}_{i,:,:,:}},b_{i}^{\prime }={{\beta }_{i}}-\frac{{{\mu }_{i}}{{\gamma }_{i}}}{{{\sigma }_{i}}}. \end{aligned}$$

**Fig. 3:**
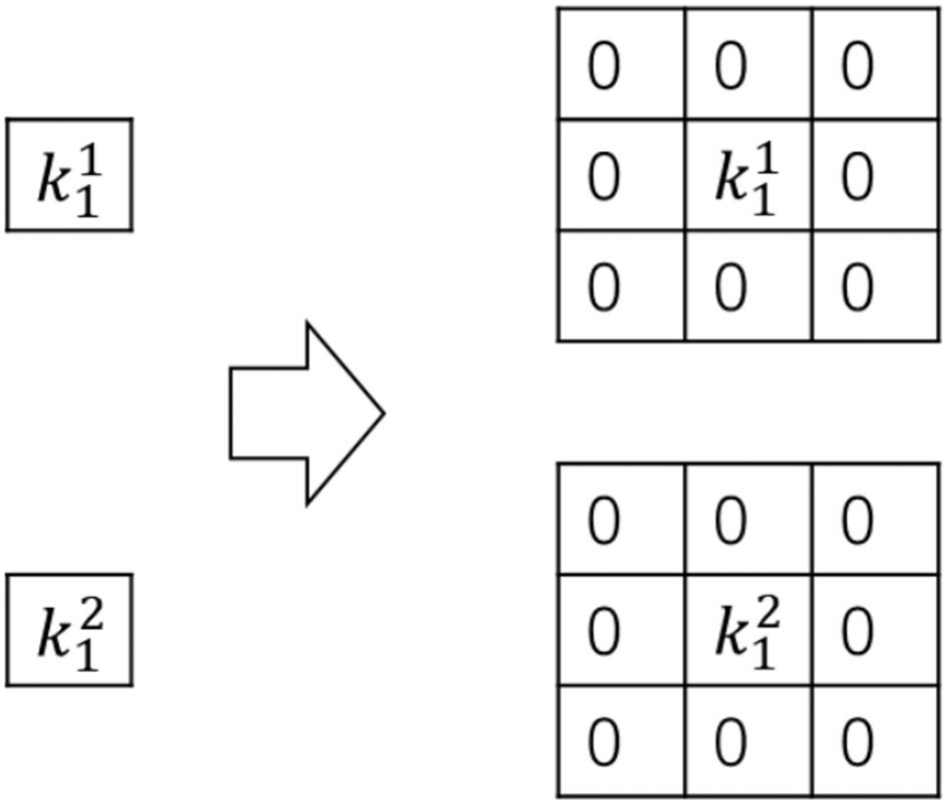
Diagram of converting a 1$\times$1 convolution to a 3$\times$3 convolution.

The 1$\times$1 convolution can be transformed into a 3$\times$3 convolution by adding zeros around the original weights, as depicted in Fig. 3. This conversion results in a 3$\times$3 convolutional layer.

**Fig. 4:**
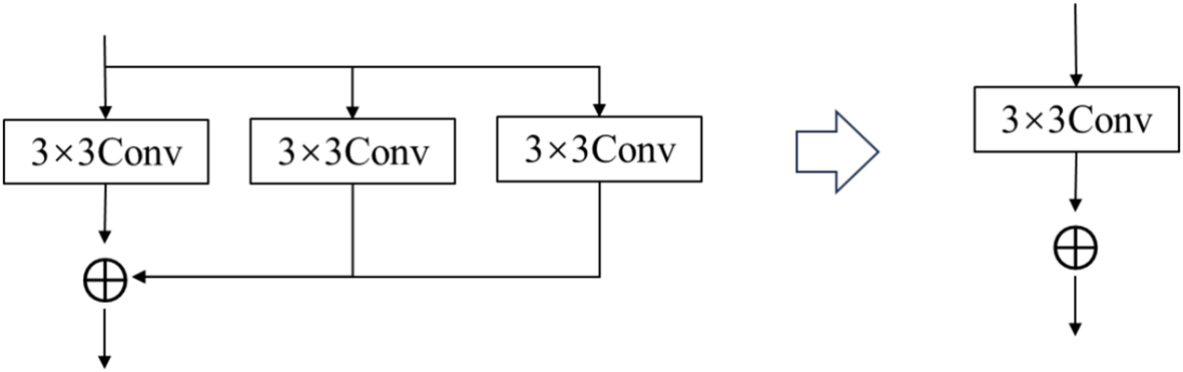
Reparameterization process of multi-branch 3$\times$3 convolutional networks.

When all branches in the network consist of 3$\times$3 convolutions, as shown in Fig. 4, the addition operations performed after branching can be combined. By summing the trained biases and weights together and then reverting to a single convolution, the single-branch network can achieve results consistent with the multi-branch network, thereby improving inference speed.

### Network structure of the ECB module

**Fig. 5:**
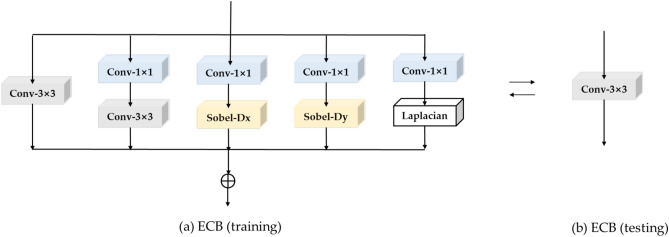
ECB network structure during training and testing.

As described in Section 2.2, multi-branch convolutions can be fused into a single convolution during inference. Inspired by ECBSR^20^, this paper adopts the ECB module from ECBSR, using a multi-branch structure during training and merging it into a single-branch network during inference to enhance inference speed. The ECB module is illustrated in Fig. 5:

The ECB module, depicted in Fig. 5(a), comprises four types of operators.

A 3$\times$3 convolution: It initially utilizes a standard 3$\times$3 convolution to ensure basic performance. This conventional convolutional operation expressed as:

9 $$\begin{aligned} {{{F}}_{{n}}}\text {=}{{{K}}_{{n}}}\times {X+}{{{B}}_{{n}}}. \end{aligned}$$

Where ${{F}_{n}}\text {, }X\text {, }{{K}_{n}}$ and ${{B}_{n}}$ respectively represent the output features, input features, weights, and biases of the standard convolution.

Dilated-Compress Convolution Combination: Wider features significantly enhance expressiveness and contribute to better performance in SR tasks. As the second component of the ECB, dilated-compress convolution is utilized. As depicted in Fig. 5(a)’s second column, it starts with a 1$\times$1 convolution as the dilated convolution, doubling the number of channels to enhance expressiveness. Subsequently, a 3$\times$3 convolution is employed as the compress convolution to restore the number of channels.

Using $\{{K}_{e}\text {, }{B}_{e}\}$ and $\{{K}_{s}\text {, }{B}_{s}\}$ to represent the weights and biases of the 1$\times$1 dilated convolution and 3$\times$3 compress convolution, respectively, the dilated-compress convolution is expressed as follows in Eq. (10):

10 $$\begin{aligned} {{F}_{es}}={{K}_{s}}\times \left( {{K}_{e}}X+{{B}_{e}} \right) +{{B}_{s}}. \end{aligned}$$

Convolution with scaled Sobel filters: Edge information has been proven to be highly beneficial for SR task^24^. ECB incorporates the extraction of first-order derivatives into its design. Due to the challenge of automatically learning sharp edge filters, ECB opts to use predefined edge filters and learns scaling factors for each filter. Specifically, the input features undergo a standard 1$\times$1 convolution first, followed by the utilization of two scaled Sobel filters to extract the gradients of the intermediate features.

Let ${{D}_{x}}$ and ${{D}_{y}}$represent the horizontal and vertical Sobel filters, respectively. They are expressed as shown in Eq. (11):

11 $$\begin{aligned} {{{D}}_{{x}}}{=}\left[ \begin{array}{lll} \text {+1} & \text {0} & \text {-1} \\ \text {+2} & \text {0} & \text {-2} \\ \text {-1} & \text {0} & \text {-1} \\ \end{array} \right] \text {, }{{{D}}_{{y}}}{=}\left[ \begin{array}{lll} \text {+1} & \text {+2} & \text {+1} \\ \text {0} & \text {0} & \text {0} \\ \text {-1} & \text {-2} & \text {-1} \\ \end{array} \right] \text {.} \end{aligned}$$

The extraction of edge information in both horizontal and vertical directions for each channel of the intermediate features involves processing with Sobel filters followed by scaling according to channel-specific scaling factors. The extraction of edge information in the horizontal and vertical directions is represented as shown in Eqs. (12) and (13):

12 $$\begin{aligned} & {{F}_{{{D}_{x}}}}=\left( {{S}_{{{D}_{x}}}}\cdot {{D}_{x}} \right) \otimes \left( {{K}_{x}}\times X+{{B}_{x}} \right) +{{B}_{{{D}_{x}}~}}. \end{aligned}$$

13 $$\begin{aligned} & {{F}_{{{D}_{y}}}}=({{S}_{{{D}_{y}}}}\cdot {{D}_{y}})\otimes ({{K}_{y}}\times X+{{B}_{y}})+{{B}_{{{D}_{y}}}}. \end{aligned}$$

Where $\{{K}_{x}\text {, }{B}_{x}\}$ and $\{{K}_{y}\text {, }{B}_{y}\}$ represent the weights and biases of the 1$\times$1 convolutions for the horizontal and vertical branches, respectively. $\{{S}_{{{D}_{x}}}\text {, }{B}_{{D}_{x}}\}$ and $\{{S}_{{{D}_{y}}}\text {, }{B}_{{{D}_{y}}}\}$ denote the scaling parameters and biases. The edge information extracted by the horizontal and vertical Sobel filters is directly summed to obtain the combined edge information ${{F}_{sob}}$, as shown in Eq. (14):

14 $$\begin{aligned} {{{F}}_{{sob}}}{=}{{{F}}_{{{{D}}_{{x}}}}}{+}{{{F}}_{{{{D}}_{{y}}}}}. \end{aligned}$$

Convolution with a combination of scaled Laplacian filters: In addition to first-order derivatives, the ECB module also employs Laplacian filters to extract second-order spatial derivatives. The input features first undergo a standard 1$\times$1 convolution, followed by the extraction of second-order spatial derivatives using a Laplacian filter ${{D}_{lap}}$, which is represented as shown in Eq. (15):

15 $$\begin{aligned} {{D}_{lap}}=\left[ \begin{matrix} 0 & +1 & 0 \\ +1 & -4 & +1 \\ 0 & +1 & 0 \\ \end{matrix} \right] . \end{aligned}$$

The extraction of scaled second-order edge information is represented as shown in Eq. (16):

16 $$\begin{aligned} {{F}_{lap}}=({{S}_{lap}}\cdot {{D}_{lap}})\otimes ({{K}_{l}}\times X+{{B}_{l}})+{{B}_{lap}}. \end{aligned}$$

Where $\{{K}_{l}\text {, }{B}_{l}\}$ represent the weights, biases of the 1$\times$1 convolution, and $\{{S}_{lap}\text {, }{B}_{lap}\}$ are the scaling factors and biases of ${{D}_{lap}}$. The output of ECB consists of four parts:

17 $$\begin{aligned} F={{F}_{n}}+{{F}_{es}}+{{F}_{sob}}+{{F}_{lap}}. \end{aligned}$$

Then, the combined feature map is passed through a non-linear activation layer, specifically a Parametric Rectified Linear Unit (PReLU).

### Network structure of the REPLKA module

**Fig. 6:**
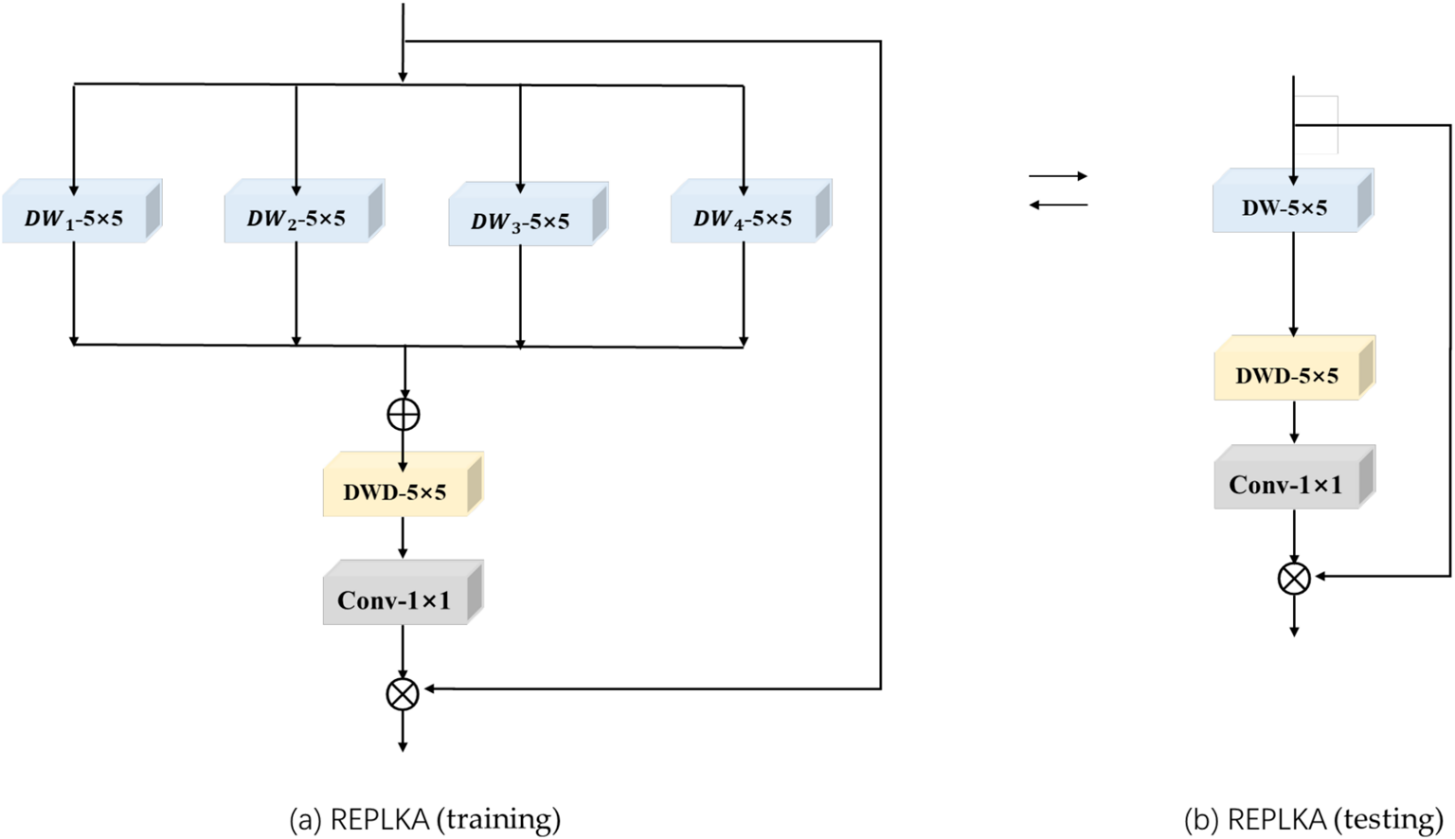
REPLKA module network structure during training and testing.

Applying large convolutional kernels in networks can increase their receptive field without reducing the effective resolution of features. However, these kernels may introduce blank regions during the convolution process, leading to the loss of local information. To effectively capture information from the input feature maps, it is necessary to employ multiple large convolutional kernels in parallel. By adopting reparameterization strategies, the model’s ability to extract information can be enhanced without increasing computational costs. Inspired by RepVGG and ECBSR, this paper introduces for the first time the strategy of large kernel reparameterization. Based on large kernel reparameterization, the REPLKA module is proposed. The network structure of REPLKA is depicted in Fig. 6. The process during training of the REPLKA module can be represented as follows:

18 $$\begin{aligned} \left\{ \begin{matrix} Y & = & D{{W}_{1}}(X)D{{W}_{2}}\left( X \right) +D{{W}_{3}}\left( X \right) +D{{W}_{4}}(X) , \\ Z & = & DW{{D}_{5\times 5}}(Y), \\ Z & = & Con{{v}_{1\times 1}}(Z) ,\\ Z & = & Z\otimes X . \\ \end{matrix} \right. \end{aligned}$$

Where $D{{W}_{1}}\left( \cdot \right)$, $D{{W}_{2}}\left( \cdot \right)$, $D{{W}_{3}}\left( \cdot \right)$, $D{{W}_{4}}\left( \cdot \right)$ denote four depthwise convolutions with kernel size of 5$\times$5, aiming to enhance the model’s expressive capacity. $DWD_{5\times 5}\left( \cdot \right)$ represents a depthwise dilated convolution with kernel size of 5$\times$5 and dilation rate of 3. $C\text {on}{{\text {v}}_{1\times 1}}\left( \cdot \right)$ denotes a convolution with kernel size of 1$\times$1. $\otimes$ denotes element-wise multiplication.

During inference, the biases and weights trained in the processes of $D{{W}_{1}}\left( \cdot \right)$, $D{{W}_{2}}\left( \cdot \right)$, $D{{W}_{3}}\left( \cdot \right)$ and $D{{W}_{4}}\left( \cdot \right)$ can be summed together. The resulting biases and weights are then used as the biases and weights in the depthwise convolutions during inference. The specific process is illustrated in Eq. (19):

19 $$\begin{aligned} \left\{ \begin{matrix} K_d=K_1+K_2+K_3+K_4, \\ B_d=B_1+B_2+B_3+B_4. \end{matrix}\right. \end{aligned}$$

Where $\left\{ {{K}_{1}},{{K}_{2}},{{K}_{3}},{{K}_{4}} \right\}$ and $\{{{B}_{1}},{{B}_{2}},{{B}_{3}},{{B}_{4}}\}$ represent the weights, biases of $D{{W}_{1}}\left( \cdot \right)$, $D{{W}_{2}}\left( \cdot \right)$, $D{{W}_{3}}\left( \cdot \right)$ and $D{{W}_{4}}\left( \cdot \right)$. ${{K}_{d}}$ and ${{B}_{d}}~$denote the weights and biases in the depthwise convolutions during inference.

The pseudocode of the proposed method in this paper is presented in Table 1.

**Table 1:** Pseudocode of the REPLKASR network.

Component	Operation
**Input**	Low-resolution image tensor $I_{LR} \in \mathbb {R}^{B \times 3 \times H \times W}$
**shallow feature extraction**	$\text {shallow feature }F_p \leftarrow \text {ECB}(I_{LR})$
**Backbone processing**	$$\begin{aligned}&F_0 = F_p \\&\textbf{for}\ i=1\ \textbf{to}\ n-1\ \textbf{do} \\&\quad F_i \leftarrow \text {ECB}_i(F_{i-1}) \\&F_n \leftarrow \text {REPLKA}(F_{n-1}) \\&F_r \leftarrow \text {ECB}_n(F_n) \\ \end{aligned}$$
**Residual fusion**	$\text {feats} \leftarrow F_p + F_r$
**Upsampling**	$I_{SR} \leftarrow \text {PixelShuffle}(\text {feats})$
	*// Sub-pixel convolution with scale factor S*
**Output**	High-resolution image $I_{SR} \in \mathbb {R}^{B \times 3 \times H{\times }S \times W{\times }S}$

## Experiments

### Experimental setup

Because acquiring infrared data is challenging, this chapter adopts a transfer learning strategy. Initially, the RepLKASR network is trained using 2650 pairs of visible images from Flickr2K^25^ and 800 pairs of visible images from DIV2K^26^.

Subsequently, 100 infrared images are selected from the M3FD^27^ public infrared dataset, denoted as M3FD-100. Among these, 70 images are used for training, 15 for validation, and 15 for testing (referred to as M3FD-15). Additionally, 15 images are chosen from the Iray infrared super-resolution dataset^28^ for testing (referred to as Iray-15). Furthermore, 15 images each are selected from the Iray infrared boat target recognition dataset (abbreviated as Iray-boat) and the Iray infrared ship traffic dataset (abbreviated as Iray-traffic) for testing. All the aforementioned infrared images are generated using bicubic degradation to form paired data.

In addition to the aforementioned datasets, this section includes 15 infrared images of traffic scenes captured around the campus for no-reference testing, named as “self-built”. For data augmentation in the training dataset, random combinations of rotations ($0^\circ$, $90^\circ$, $180^\circ$, $270^\circ$) and horizontal flips are applied. The evaluation metrics utilize the average PSNR and SSIM on the luminance channel.

In RepLKASR, this chapter employs $n=$8 ECBs and 1 REPLKA, with a channel width set to 32.

The model is trained using the Adam^29^ optimizer with parameters $\beta$1=0.9 and $\beta$2=0.99. The learning rate is initialized to 5e-4 and scheduled using cosine annealing throughout the entire training process of 1e6 iterations. For ablation studies, all models are trained within 4e5 iterations. The exponential moving average (EMA)^30^ weight is set to 0.999. Only L1 loss is utilized for optimizing the model. The patch size and batch size for RepLKASR are set to 192$\times$192 and 64, respectively. The same training strategy is applied during transfer learning. The compared methods in this chapter also undergo transfer learning using the same strategy.

The experiments in this section are conducted using the Ubuntu 20.04 operating system and the PyTorch 1.9.0 training framework.

### Ablation experiment of the REPLKA module

To verify the effectiveness of the proposed large kernel reparameterization module in REPLKASR, ablation experiments are conducted in this section. To conserve computational resources, the ablation experiments are uniformly trained for 400,000 iterations. The performance of the model is evaluated on benchmark datasets including Set5^31^, Set14^32^, BSD100^33^, Urban100^34^, and Manga109^35^.

As shown in Table 2, training with the four-branch module achieves better performance. During inference, the reparameterization strategy ensures that the computational complexity of the four-branch network matches that of the single-branch network, demonstrating the effectiveness of the proposed REPLKA module.

From “Single-Branch” to “Four-Branch,” as the number of branches increases, the PSNR and SSIM values gradually improve on different datasets. For example, on the Set5 dataset, the PSNR/SSIM increases from 31.60/0.8867 to 31.66/0.8882, indicating that more branches contribute to improving the image quality.

In the “Multi-Adds (G)” column, it can be seen that the computational complexity of all configurations containing REPLKA modules is 6.6 GOPs (Giga Operations), indicating that the network’s total computational complexity remains unchanged despite the addition of REPLKA module branches. This is because the structural reparameterization method optimizes the internal structure to maintain computational efficiency.

**Table 2:** Ablation Study of the REPLKA Module: The impact of different configurations, including the absence of the REPLKA module, single-branch module, two-branch module, three-branch module, and four-branch module, on $\times$2 Super-Resolution tasks performed by REPLKASR is investigated. The best metrics are highlighted in bold.

REPLKA Module	Scale	Params (K)	Multi- Adds (G)	Set5 PSNR/SSIM	Set14 PSNR/SSIM	BSD100 PSNR/SSIM	Urban100 PSNR/SSIM	Manga109 PSNR/SSIM
No REPLKA	$\times$2	111	6.4	31.60/0.8867	28.27/0.7738	27.34/0.7279	25.38/0.7613	29.47/0.8943
Single- Branch		113	6.6	31.63/0.8873	28.29/0.7745	27.35/0.7284	25.45/0.7641	29.57/0.8957
Two- Branch		113	6.6	31.64/0.8877	28.30/0.7747	27.34/0.7286	25.45/0.7643	29.57/0.8959
Three- Branch		113	6.6	**31.66**/0.8877	28.30/**0.7749**	27.36/0.7288	25.45/0.7645	29.60/0.8958
Four- Branch		113	6.6	**31.66/0.8882**	**28.31/0.7749**	**27.36/0.7290**	**25.46/0.7647**	**29.62/0.8960**

**Fig. 7:**
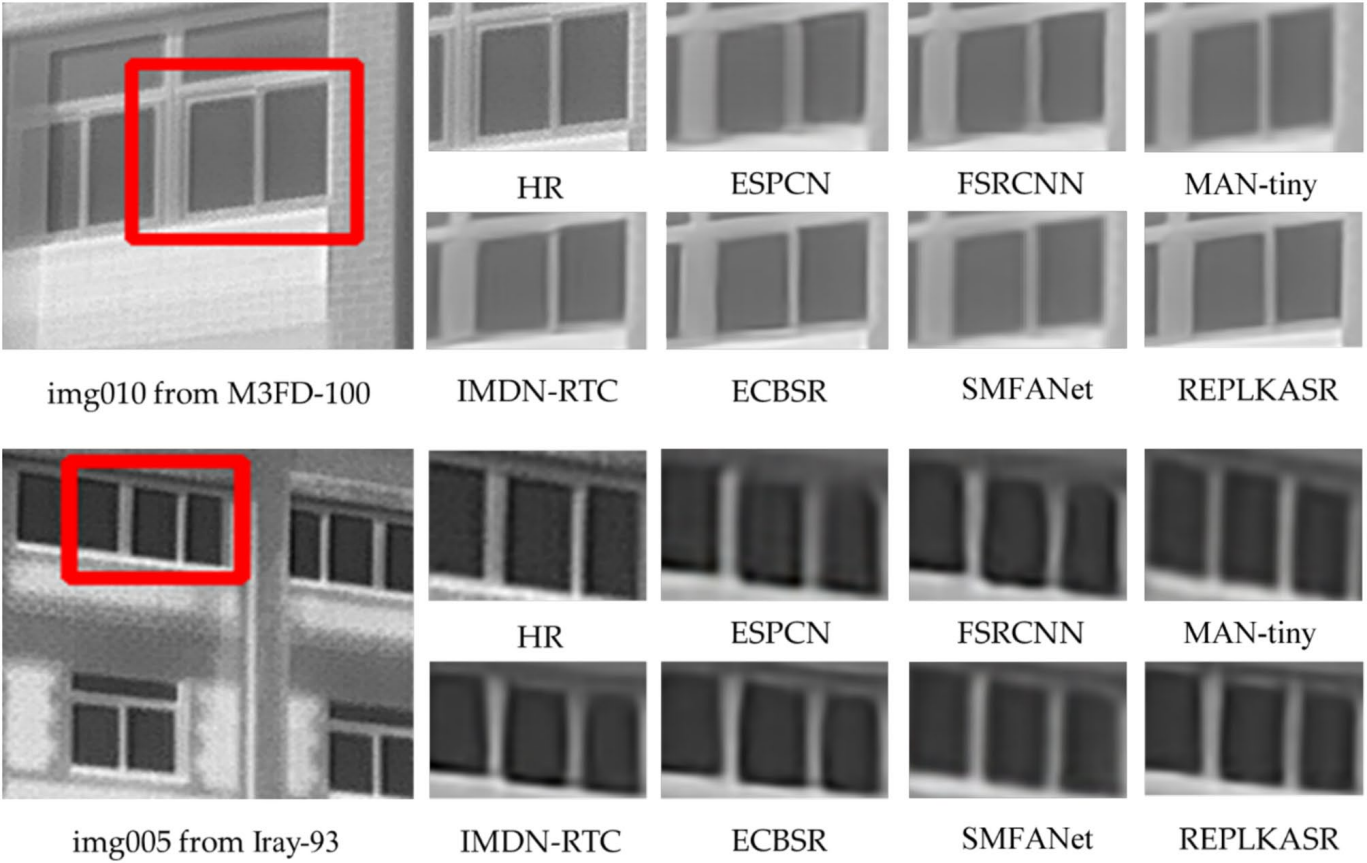
Visual comparison with State-of-the-Art methods on Challenging Cases in M3FD-15 and Iray-15 datasets ($\times$4 Super-Resolution).

### Results of the infrared image experiment

Since there are few dedicated lightweight models for infrared image super-resolution reconstruction, this paper evaluates the performance of the proposed RepLKASR by conducting transfer learning with the same infrared data using classical lightweight SR methods with comparable parameters. The methods compared include ESPCN^36^, FSRCNN^37^, IMDN-RTC^38^, ECBSR-M10C32^20^, MAN-tiny^39^ and SMFANet^40^. Table 4 presents the quantitative comparison on the M3FD-15, Iray-15, Iray-boat and Iray-traffic datasets with an upsampling factor of $\times$4. Both tables also provide the Params, Multi-Adds and FLOPs for an output resolution of 1280$\times$720.

**Table 3:** Quantitatively compare with the state-of-the-art methods in the image super-resolution field on the Infrared Images benchmark dataset. The best and second-best performances are highlighted in Italic and bold, respectively.

Method	Scale	Params (K)	Multi- Adds (G)	FLOPs (G)	M3FD-15 PSNR/SSIM	Iray-15 PSNR/SSIM	Iray-boat PSNR/SSIM	Iray-traffic PSNR/SSIM
ESPCN	$\times$2	21	2.28	4.55	30.44/0.8820	33.86/0.9123	30.83/0.9031	31.50/0.9125
FSRCNN		12	3	6	30.75/0.8879	34.21/0.9200	31.11/0.9138	31.81/0.9214
IMDN-RTC		19	2.29	4.57	30.92/0.8920	34.45/0.9226	31.30/0.9164	32.00/0.9237
ECBSR		94	10.9	21.8	**31.29**/0.8956	**34.65**/0.9244	**31.48/0.9187**	**32.28/0.9262**
MAN-tiny		150	4.2	8.4	31.17/*0.9018*	34.61/*0.9272*	31.35/0.9157	31.98/0.9219
SMFANet		186	20.5	41	31.13/**0.9016**	34.55/**0.9268**	31.34/0.9157	31.93/0.9218
REPLKASR		105	21.2	42.4	*31.31*/0.8964	*34.73*/0.9247	*31.49/0.9189*	*32.33/0.9266*
ESPCN	$\times$4	24	0.72	1.44	25.06/0.6906	27.90/0.7921	25.76/0.7542	26.43/0.7889
FSRCNN		12	2.3	4.6	25.13/0.6954	28.01/0.7974	25.79/0.7589	26.48/0.7928
IMDN-RTC		21	0.61	1.22	25.12/0.6990	28.05/0.8013	25.81/0.7611	26.47/0.7947
ECBSR		98	2.83	5.65	25.14/**0.7000**	28.11/**0.8038**	25.84/**0.7628**	26.49/**0.7964**
MAN-tiny		150	4.2	8.4	**25.16**/0.6945	28.16/0.7976	**25.88**/0.7497	**26.52**/0.7802
SMFANet		197	5.5	11	*25.17*/0.6955	*28.19*/0.7990	25.87/0.7495	**26.52**/0.7808
REPLKASR		113	6.6	13.2	*25.17/0.7010*	**28.17**/*0.8045*	*25.91/0.7641*	*26.53/0.7968*

**Fig. 8:**
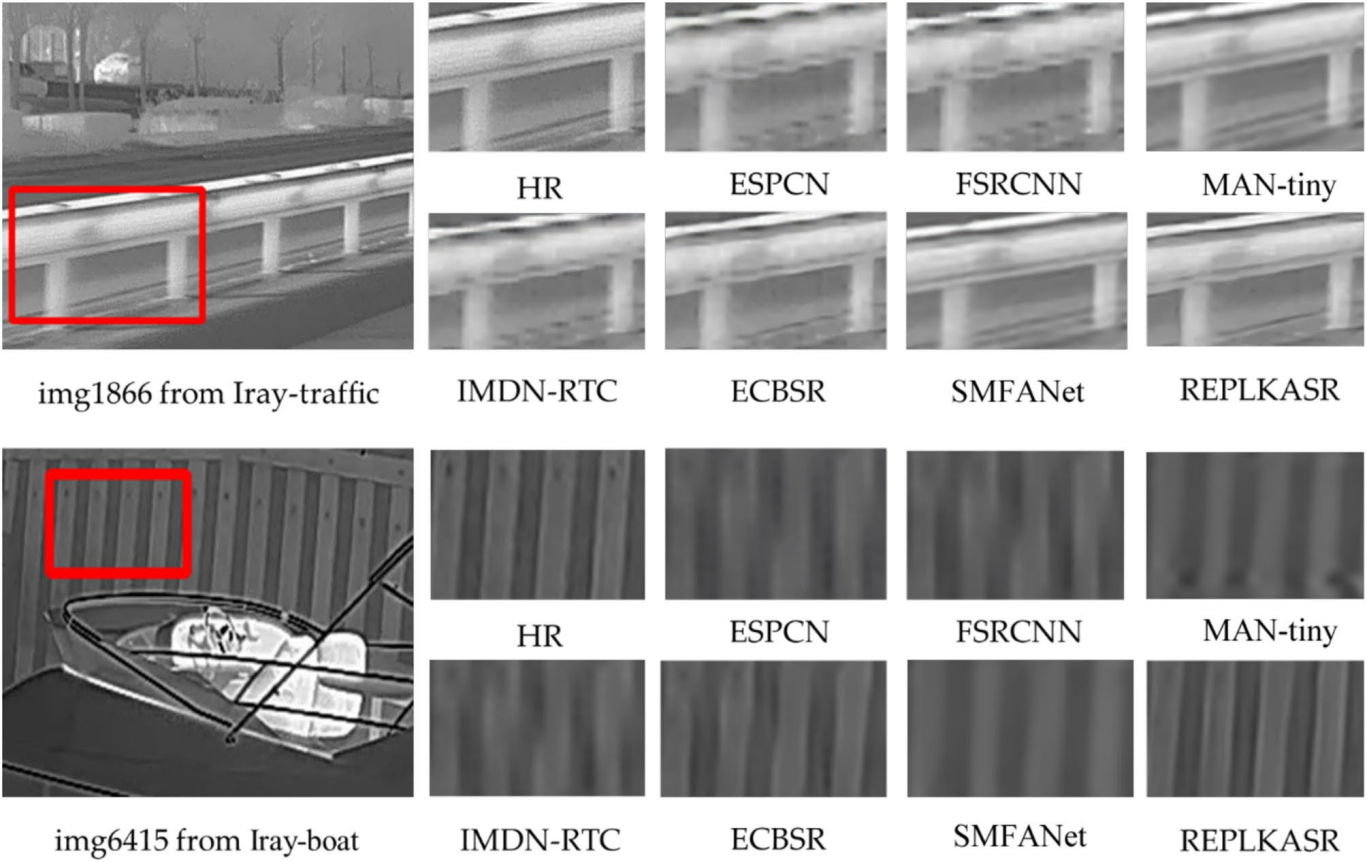
Visual comparison with State-of-the-Art Methods on Challenging Cases in Iray-boat and Iray-traffic Datasets ($\times$4 Super-Resolution).

As shown in Table 3, REPLKASR demonstrates significant advantages in multiple key metrics. It achieves the best or second-best performance in terms of PSNR and SSIM for both $\times$2 and $\times$4 upscaling factors. Particularly, at the $\times$4 upscaling factor, REPLKASR achieves the highest PSNR and SSIM values on the M3FD-15, Iray-boat, and Iray-traffic datasets. Although REPLKASR slightly surpasses some methods in terms of parameter count and computational complexity (Multi-Adds and FLOPs), its significant improvement in image quality proves its efficiency and superiority in infrared image super-resolution tasks. Therefore, REPLKASR, while maintaining high computational efficiency, can provide higher-quality super-resolution images, demonstrating important practical value.

**Fig. 9:**
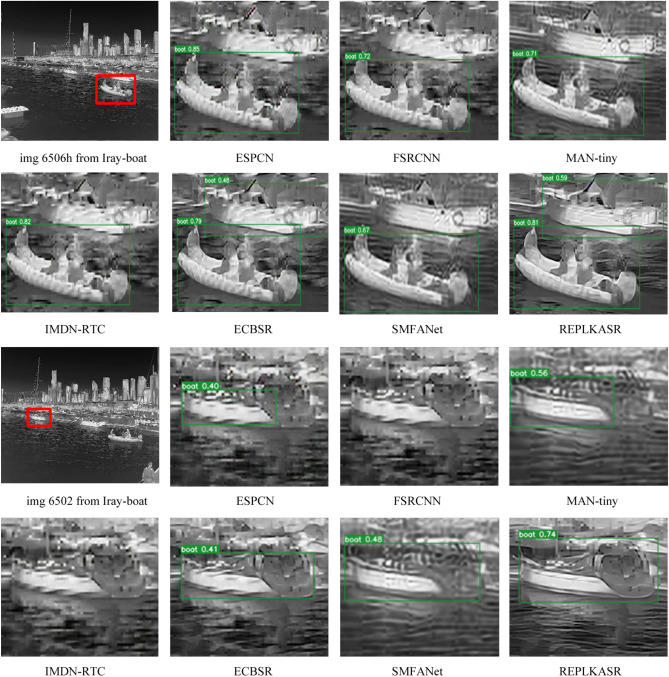
Visual Comparison and YOLOv5 Object Detection Results of $\times$4 Super-Resolution in Challenging Scenarios on the Iray-boat Dataset with State-of-the-Art Methods.

**Fig. 10:**
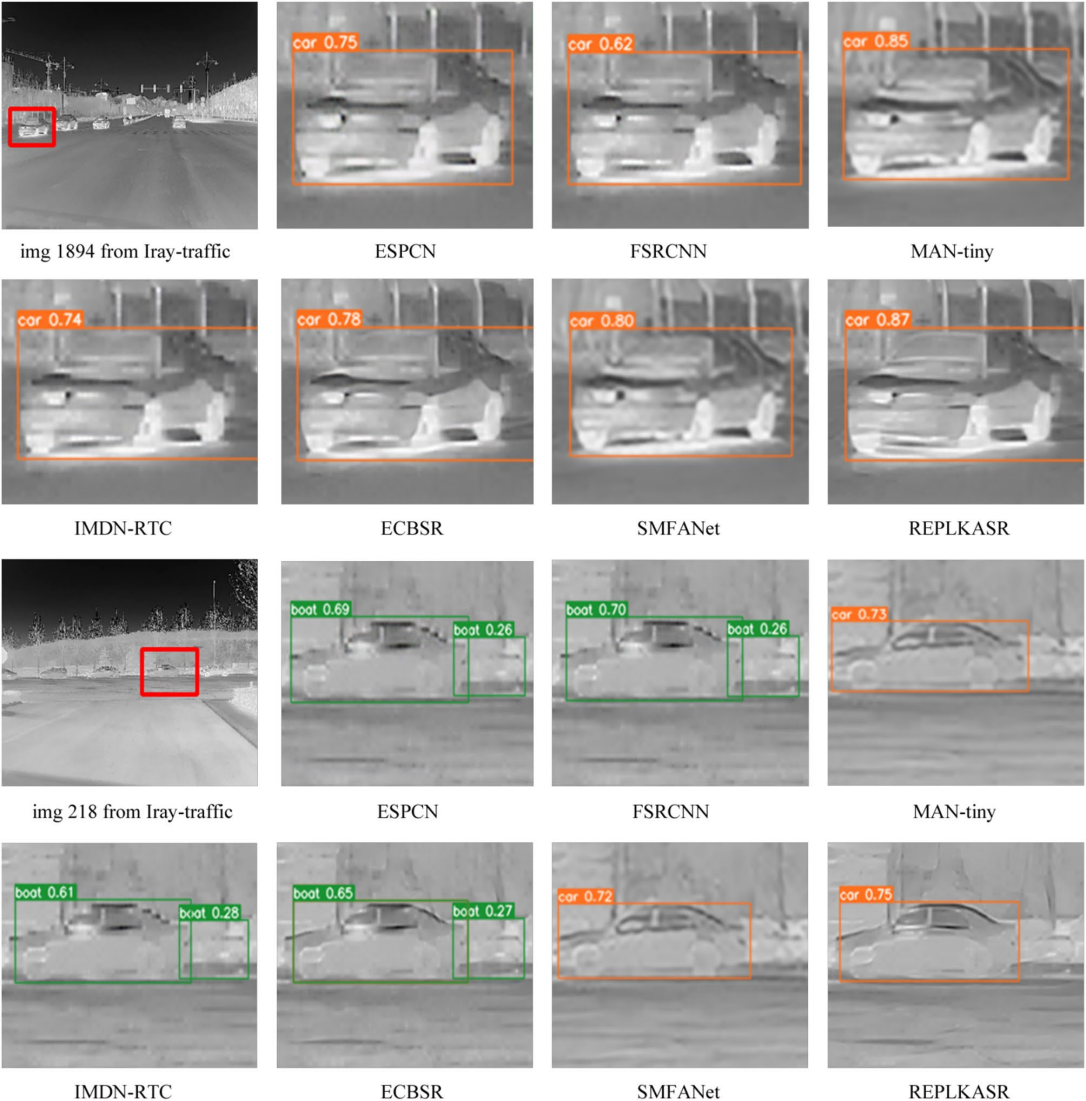
Visual Comparison and YOLOv5 Object Detection Results of $\times$4 Super-Resolution in Challenging Scenarios on the Iray-traffic Dataset with State-of-the-Art Methods.

In addition to quantitative evaluations, visual comparisons between the proposed RepLKASR and six state-of-the-art lightweight SR methods, including ESPCN, FSRCNN, IMDN-RTC, and ECBSR, are provided. Figures 7 and 8 present visual comparisons on the $\times$4 M3FD-15 dataset and Iray-15 dataset with the state-of-the-art methods. The images within the red boxes are cropped and magnified. Figures 9 and 10 show visual comparisons on the $\times$4 Iray-boat and Iray-traffic datasets, respectively. To further demonstrate the effectiveness of the proposed RepLKASR, the confidence levels detected by YOLOv5 are also presented in Figs. 9 and 10.

In Fig. 7, for img010 in the M3FD-15 dataset and img1866 in the Iray-traffic dataset, the proposed RepLKASR method can restore the window image to a level almost indistinguishable from the HR image, while other methods still produce images with blur and artifacts, failing to restore straight lines, resulting in unacceptable reconstructions. In Fig. 8, for img005 in the Iray-15 dataset and img6415 in the Iray-boat dataset, the image restored by the proposed RepLKASR method is clear and clean, while the reconstructions by other methods exhibit broken contours or distorted contours, leading to unacceptable results.

In the visual results of Figs. 7 and 8, the proposed method achieves the best visual effects among the results, and the results of the ECBSR method come closest to those of the proposed method. This is because ECBSR utilizes multiple parallel small kernel convolution modules, known as ECB. In contrast, the proposed method introduces large kernel convolution blocks based on the ECBSR network, which increases the receptive field for feature learning compared to ECB. As a result, the proposed method can extract more details and has a larger image restoration range compared to ECBSR.

As shown in Fig. 9 and Fig. 10, REPLKASR achieved the highest confidence scores in multiple object detection tasks. No instances of false negatives were observed in any of the test images for REPLKASR, while most other methods exhibited false negatives in images img6506 and img6502. This indicates that REPLKASR has higher robustness and reliability in object detection tasks. Additionally, REPLKASR did not exhibit any instances of false positives. This suggests that REPLKASR is able to better preserve the structural information of the objects and reduce interference from background noise when generating super-resolution images.

In conclusion, the proposed RepLKASR algorithm achieves the best super-resolution performance in various scenarios, yielding the highest target detection confidence, thus demonstrating the effectiveness of the proposed method.

### Neural network processor inference results

The neural network processor chosen for this study is the Rockchip RK3588 development board. This chip features an eight-core CPU with four A76 cores and four A55 cores, as well as an ARM G610MP4 GPU. It also includes an integrated NPU with a computational power of 6 TOPs (Tera Operations Per Second), capable of performing 6 trillion operations per second. This processor is characterized by high computational power, low power consumption, and multiple interfaces, making it well-suited to the system requirements.

**Fig. 11:**
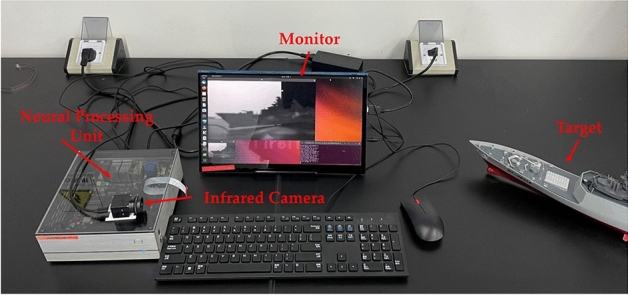
Real-time infrared super-resolution system.

Moreover, the platform runs on the Ubuntu 20.04 operating system, allowing for direct configuration of necessary code libraries and open-source deep learning frameworks, significantly simplifying the network deployment process. For the infrared sensor, the study employs the X162O-F180W uncooled infrared detector from Chengdu Jinglin, which connects via USB. This setup enables video stream reading through the software depend-ency library OpenCV (a cross-platform computer vision and machine learning software library). The real-time infrared image super-resolution system constructed in this study is illustrated in Fig. 11.

**Table 4:** Performance comparison of lightweight SR methods on the RK3588NPU. Tested on public datasets. Inference time is measured based on the output image size of 1280$\times$720. The best and second-best performances are highlighted in Italic and bold, respectively.

REPLKA Module	Scale	M3FD-15 PSNR/SSIM	Iray-15 PSNR/SSIM	Iray-boat PSNR/SSIM	Iray-traffic PSNR/SSIM	time consuming(ms)
ESPCN-uint8	$\times$4	24.76/0.6552	27.56/0.7543	30.83/0.9031	31.50/0.9125	44.24
FSRCNN-uint8		24.76/0.6616	27.74/0.7761	31.11/0.9138	31.81/0.9214	53.32
IMDN-RTC-uint8		24.78/0.6620	27.76/0.7732	31.30/0.9164	32.00/0.9237	51.67
ECBSR-uint8		**24.81/0.6656**	27.74/0.7759	**31.48/0.9187**	**32.28/0.9262**	*37.22*
REPLKASR-uint8		*24.82/0.6659*	*27.78/0.7784*	*31.49/0.9189*	*32.33/0.9266*	**37.59**

This section presents the inference speed and accuracy of the quantized REPLKASR model (abbreviated as REPLKASR-uint8) on the RK3588 platform. To further demonstrate the superiority of the proposed REPLKASR-uint8, comparisons are made with ESPCN-uint8, FSRCNN-uint8, IMDN-RTC-uint8, and ECBSR-M10C32-uint8 (abbreviated as ECBSR-uint8). Table 4 shows the accuracy and inference times of each algorithm on the RKNPU. The proposed REPLKASR-uint8 achieves real-time super-resolution for 320$\times$180 images on the RK3588NPU.

Inference results (Table 4) of the quantized algorithms on the RK3588NPU show that compared to the IMDN-RTC-uint8$\times$4 method, the proposed RepLKASR-uint8$\times$4 method achieves an average improvement of 0.05 dB in PSNR and 0.0056 in SSIM across four infrared datasets. Compared to the ECBSR-M10-uint8$\times$4, the RepLKASR-uint8$\times$4 method achieves an average improvement of 0.035 dB in PSNR and 0.0031 in SSIM across the same four infrared datasets.

In addition to the referenced dataset comparisons, we also conducted a four-fold super-resolution experiment on the self-built dataset because the real dataset lacks references. Table 5 compares the no-reference evaluation metrics, namely Natural Image Quality Evaluator (NIQE)^41^ and Perception based Image Quality Evaluator (PIQE)^42^. NIQE, based on human visual perception, indicates that a lower value corresponds to a higher visual quality of the image. PIQE is sensitive to image noise, with a lower score indicating higher image quality.

**Table 5:** Performance comparison of lightweight SR methods on the RK3588NPU. Tested on Self-built datasets. Inference time is measured based on the output image size of 1280$\times$720. The best and second-best performances are highlighted in Italic and bold, respectively.

REPLKA Module	Scale	Self-built NIQE/PIQE	time consuming(ms)
ESPCN-uint8	$\times$4	5.86/5.68	44.24
FSRCNN-uint8		5.58/5.63	53.32
IMDN-RTC-uint8		5.24/4.99	51.67
ECBSR-uint8		**4.67/4.52**	*37.22*
REPLKASR-uint8		*4.42/4.48*	**37.59**

As shown in Table 5, the proposed RepLKASR-uint8$\times$4 outperforms all compared methods in terms of NIQE and PI metrics on the self-built dataset, achieving the highest efficiency. This indicates that the proposed method in this paper outperforms other comparative methods in terms of image quality restoration, generating outputs that are closer to high-quality natural images. At the same time, the inference time is close to the optimal value, which demonstrates that the method presented in this paper achieves higher image quality while maintaining efficient inference.

**Fig. 12:**
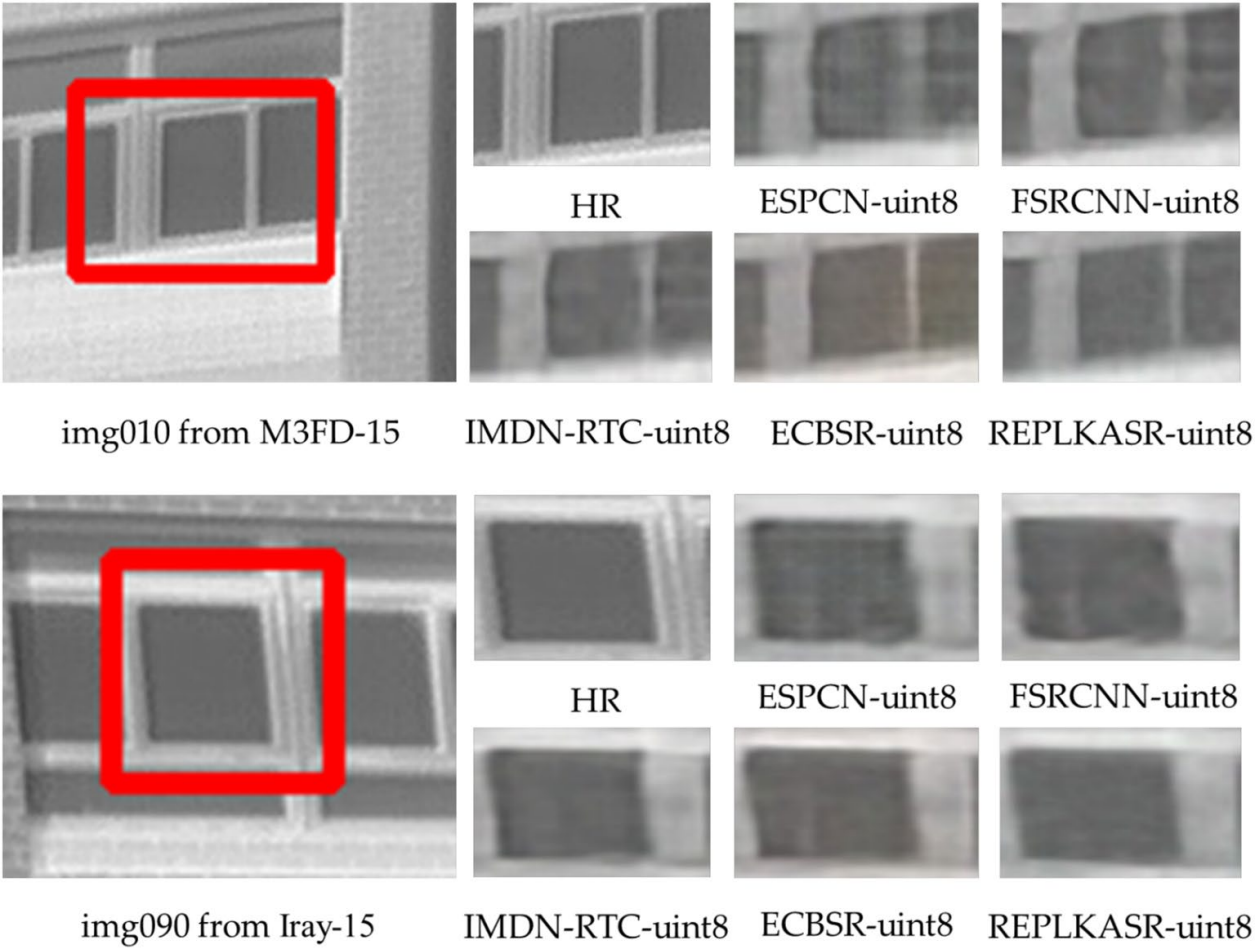
Comparison of $\times$4 Super-Resolution Results in Challenging Scenarios on the M3FD-15 and Iray-15 Datasets (RK3588NPU Inference).

**Fig. 13:**
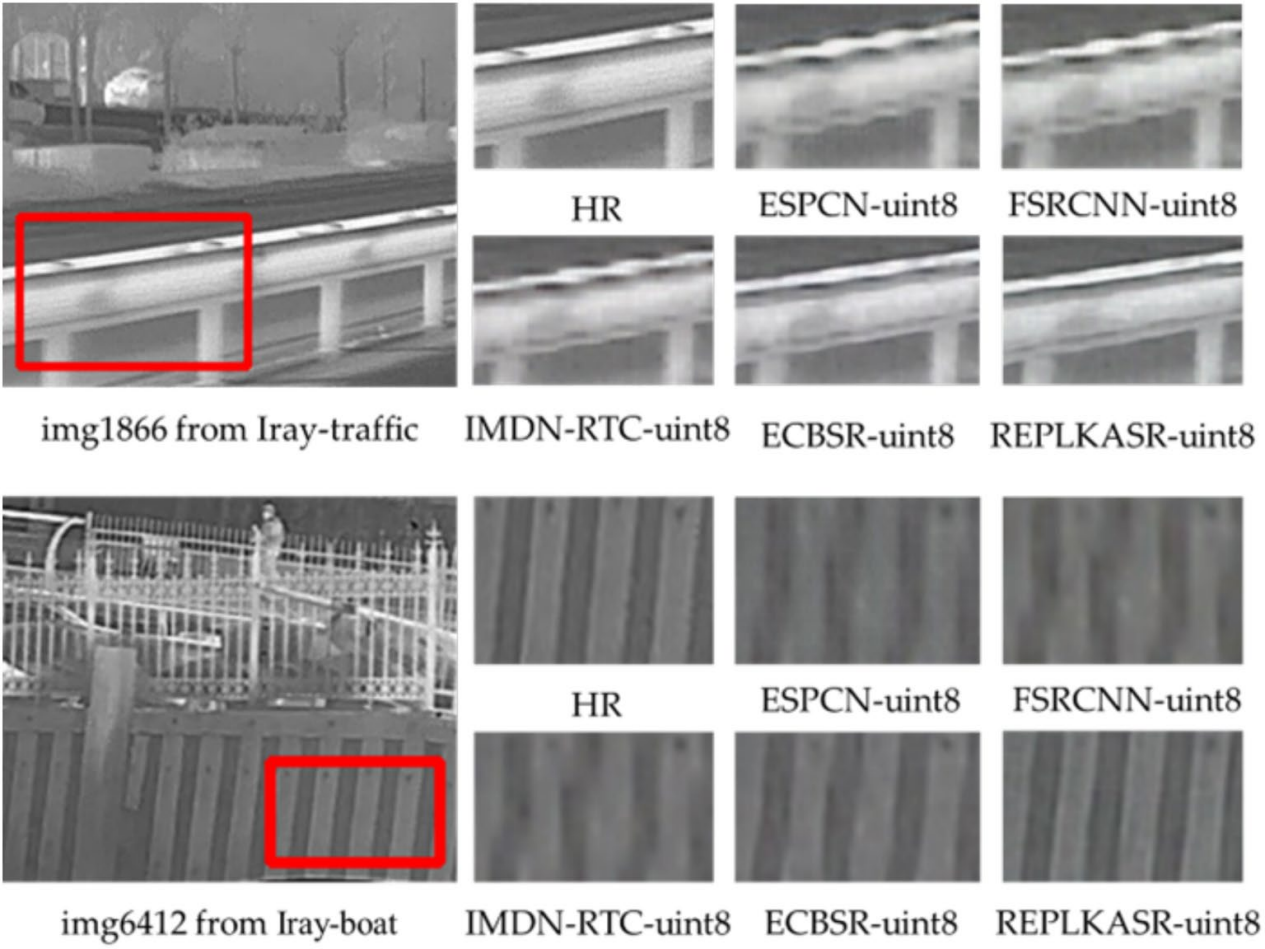
Comparison of $\times$4 Super-Resolution Results in Challenging Scenarios on the M3FD-15 and Iray-15 Datasets (RK3588NPU Inference).

In addition to quantitative evaluations, visual comparisons of the proposed RepLKASR-uint8 with state-of-the-art lightweight SR methods on the RK3588NPU are provided, including ESPCN-uint8, FSRCNN-uint8, IMDN-RTC-uint8, and ECBSR-M10-uint8. Figures 12 and 13 show visual comparisons on the four infrared datasets with $\times$4 upscaling against the state-of-the-art methods. The images in the red boxes are cropped and magnified.

**Fig. 14:**
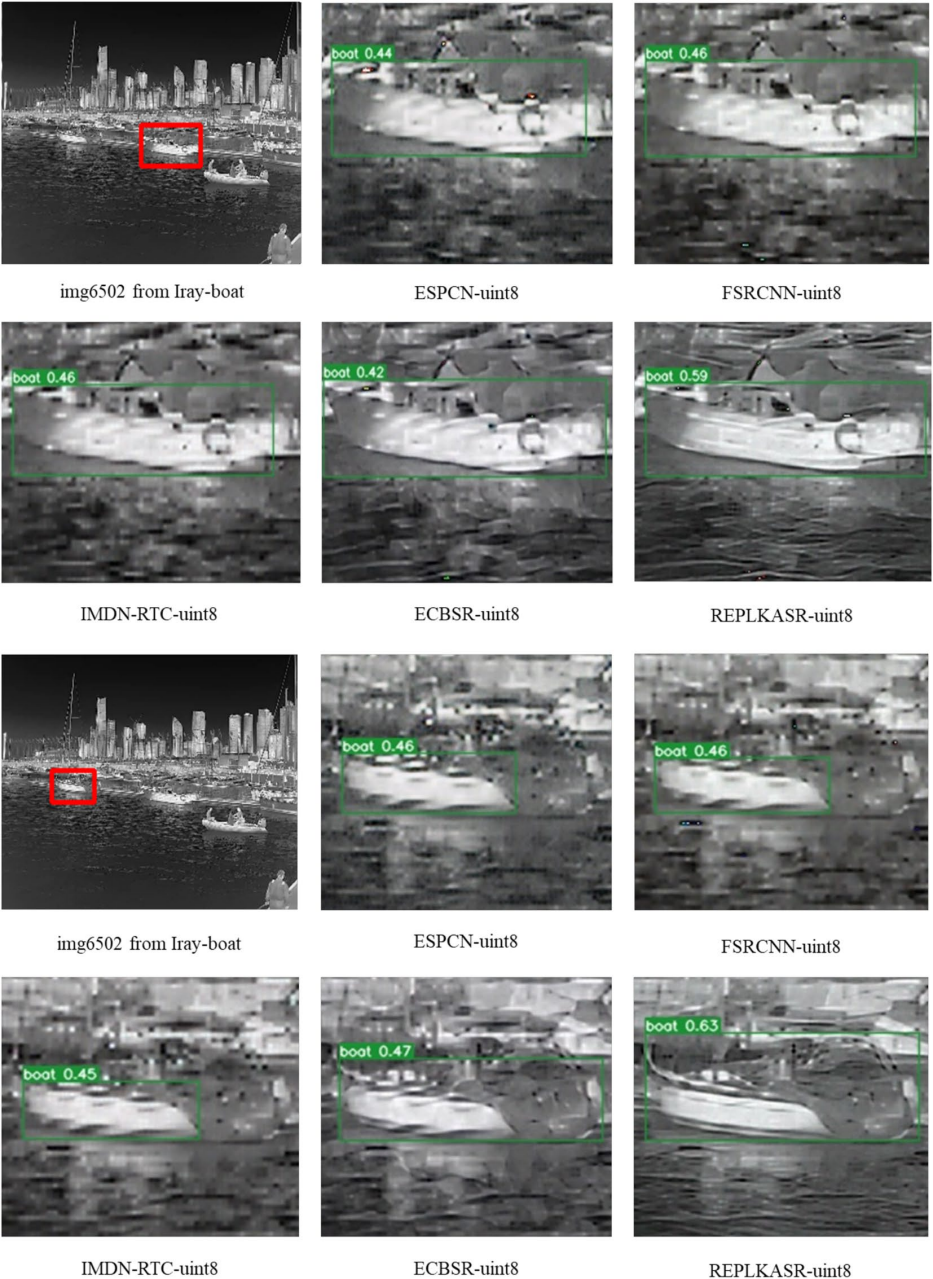
Visual comparisons under challenging scenarios on the Iray-boat dataset against state-of-the-art methods, along with yolov5 object detection results (inference on RK3588NPU) at 4$\times$ super resolution.

**Fig. 15:**
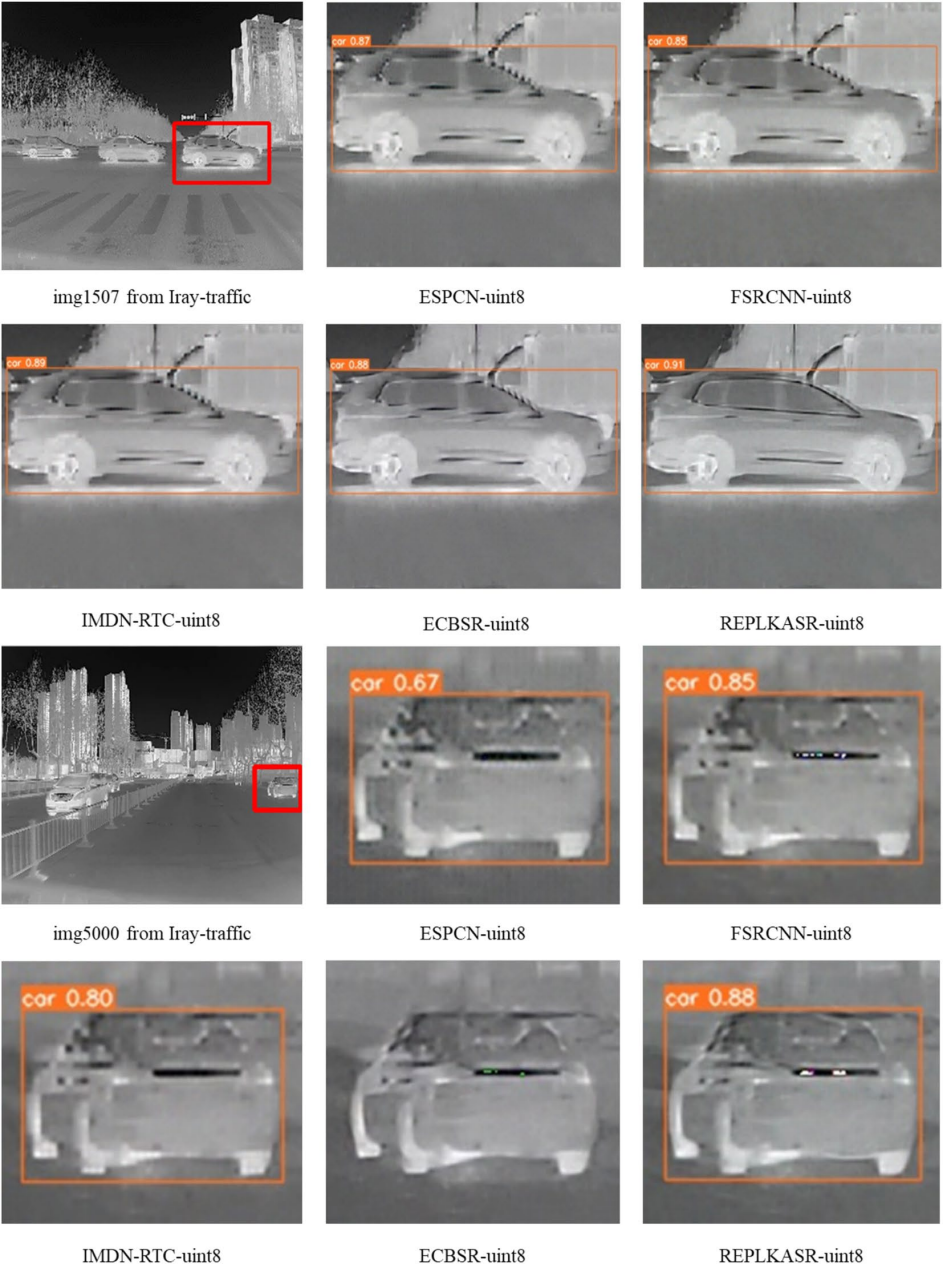
Visual comparisons under challenging scenarios on the Iray-traffic dataset against state-of-the-art methods, along with yolov5 object detection results (inference on RK3588NPU) at 4$\times$ super resolution.

**Fig. 16:**
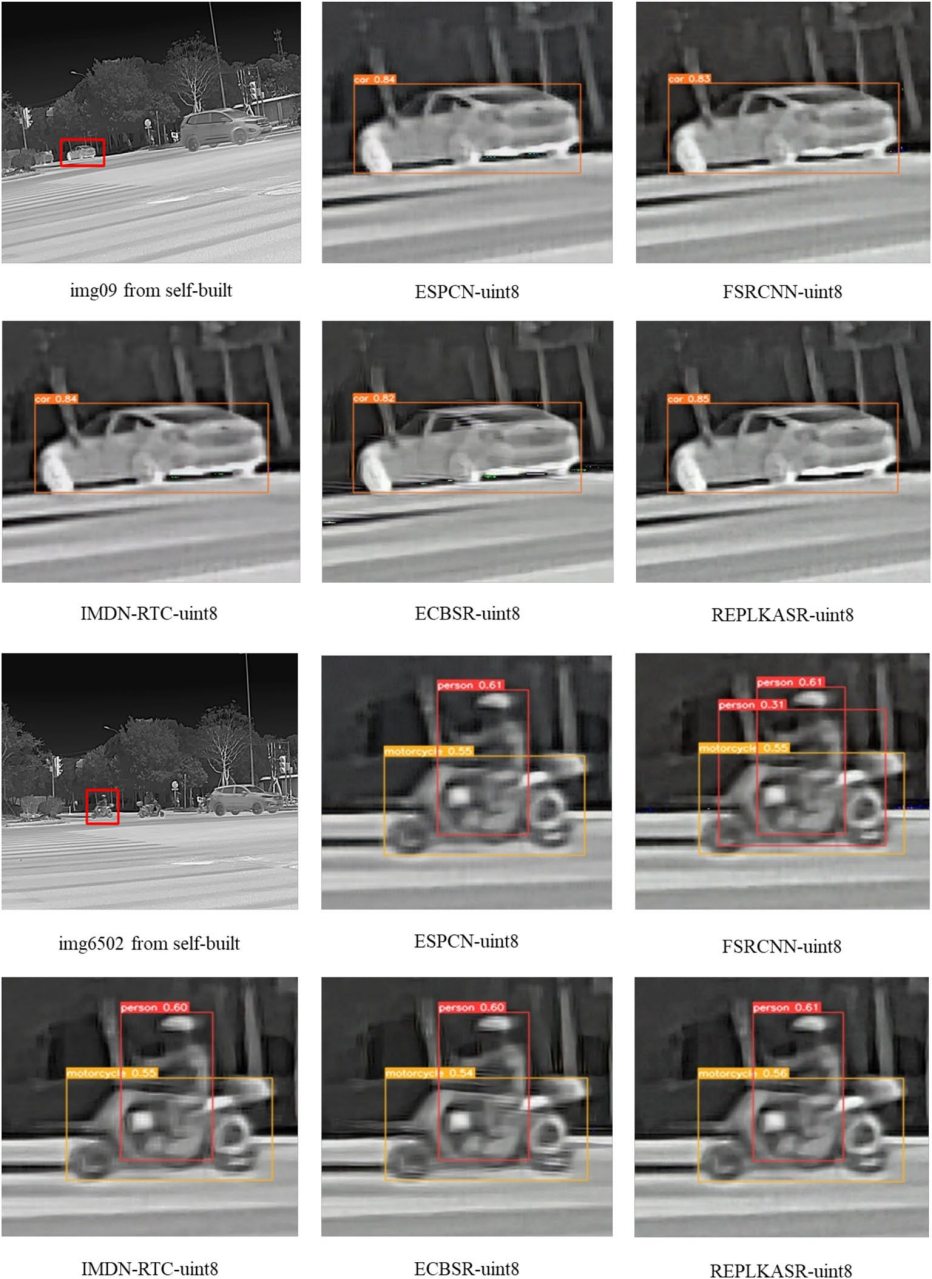
Visual comparisons under challenging scenarios on the self-built dataset against state-of-the-art methods, along with yolov5 object detection results (inference on RK3588NPU) at 4$\times$ super resolution.

Figures 14, 15, and 16 respectively display visual comparisons on the $\times$4 upscaled Iray-boat dataset, Iray-traffic dataset, and self-built dataset against the state-of-the-art methods. To further demonstrate the effectiveness of the proposed RepLKASR-uint8, the confidence scores from YOLOv5 detection are also presented.

For img1866 in Iray-traffic, the RepLKASR-uint8 method proposed can restore the fence image almost indistinguishable from HR, while other methods still produce images containing blur and artifacts, and may even fail to restore normal straight lines, resulting in unacceptable reconstruction. For img6412 in Iray-boat, the RepLKASR-uint8 method restores a clear and clean image, whereas other methods produce distorted and blurry lines, leading to unacceptable reconstruction.

The REPLKASR method performed excellently in the object detection tasks of all test images. As shown in Fig. 15 and Fig. 16, the proposed method in this paper achieved the highest confidence in vehicle detection tasks, demonstrating stronger stability and robustness. Particularly in the water scene shown in Fig. 14, REPLKASR exhibited significantly higher detection confidence for ships compared to other methods. This is because of the higher contrast between the background and the target in water scenes, and the convolutional kernels used in the REPLKASR method have a larger receptive field, allowing for more effective differentiation between the background and the target.

In summary, the RepLKASR-uint8 algorithm proposed in this study achieves superior super-resolution results in various scenarios, attaining the highest confidence in object detection. This validates the effectiveness of the proposed method.

## Discussion

### Analysis of reconstruction model limitations under noise and low-contrast conditions

There are performance variations among different datasets for each model. The proposed method in this paper showed outstanding performance on the Iray-boat and Iray-traffic datasets, which mainly focus on ship and vehicle scenes. In contrast, the performance of the models was slightly inferior on the M3FD-15 and Iray-15 datasets, which primarily consist of building and road scenes. This phenomenon may be related to the characteristics of the fine-tuning dataset (M3FD). Although the proposed method exhibited good scene generalization performance, its performance in specific scenes was not as good as models with smaller convolutional kernels.

**Fig. 17:**
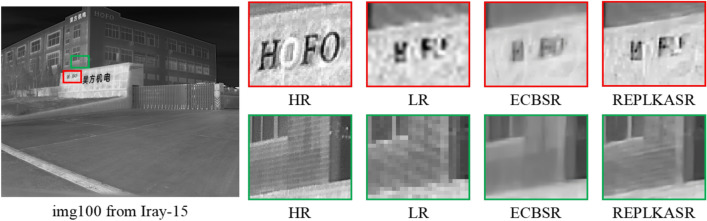
4$\times$ super-resolution reconstruction failure cases on the Iray-15 dataset.

As shown in the red box region in Fig. 17, the proposed method reconstructs the second letter “O” less effectively than the ECBSR method. This arises from the large receptive field of the kernel convolution: when the input low-resolution image contains excessive noise, the model tends to misidentify noise as valid features for reconstruction. Additionally, the first letter “O” fails to reconstruct due to insufficient contrast. Notably, in the green box region, when confronted with ambiguous information in the input image, the proposed method partially restores high-frequency details compared to ECBSR, indicating that in areas with lower noise levels, the large receptive field helps enhance reconstruction performance.

### Performance gain bottlenecks with increasing model complexity

From Table 4 and Table 5, it can be observed that as the model complexity increases, the marginal effect of performance improvement gradually diminishes. On one hand, due to the lack of high-quality infrared datasets, the models may not have been sufficiently trained, resulting in limited performance improvement. On the other hand, the increase in model complexity significantly increases the consumption of computational resources, which may limit the practicality of the model in real-time applications or resource-constrained devices. For example, at a magnification factor of $\times$2, the improvement in PSNR and SSIM metrics for SMFANet and the proposed method compared to ECBSR and MAN-tiny is not significant. This indicates that in certain cases, further increasing model complexity may not lead to significant performance improvement but instead increase the consumption of computational resources.

Therefore, in future work, we plan to explore more efficient model architectures and training strategies to reduce computational costs while maintaining or improving performance. Specifically, we consider introducing lightweight modules (such as depth-wise separable convolution) or knowledge distillation techniques to optimize model design, while enhancing model generalization and training efficiency through data augmentation or transfer learning.

## Conclusions

Currently existing infrared image super-resolution reconstruction networks often struggle to balance reconstruction performance and inference speed, making real-time processing challenging on resource-constrained edge computing platforms. Addressing this issue, this paper introduces for the first time the Large Kernel Resampling Attention Mechanism. During training, it utilizes a multi-branch large kernel network to fully extract information and converts equivalently to a single-branch large kernel network during inference, achieving a balance between processing performance and inference speed.

Compared to state-of-the-art SR methods with similar Params and FLOPs, REPLKASR improves PSNR on infrared datasets by 0.08 dB and SSIM by 0.0004. The REPLKASR model is deployed on the RK3588 neural network processor and combined with infrared addition to build a real-time super-resolution reconstruction system for infrared scenes. This system achieves four-fold real-time super-resolution for 320$\times$180 images.

For data citations of datasets uploaded to e.g. *figshare*, please use the howpublished option in the bib entry to specify the platform and the link, as in the Hao:gidmaps:2014 example in the sample bibliography file.

## Data Availability

The public data used in this work are listed here: 1. Flickr2K: https://openaccess.thecvf.com/content_cvpr_2017_workshops/w12/html/Lim_Enhanced_Deep_Residual_CVPR_2017_paper.html?ref=https://githubhelp.com; 2. DIV2K: https://openaccess.thecvf.com/content_cvpr_2017_workshops/w12/html/Agustsson_NTIRE_2017_Challenge_CVPR_2017_paper.html; 3. Set5: http://eprints.imtlucca.it/2412/; 4. Set14: https://link.springer.com/chapter/10.1007/978-3-642-27413-8_47; 5. Urban100: https://www.cv-foundation.org/openaccess/content_cvpr_2015/html/Huang_Single_Image_Super-Resolution_2015_CVPR_paper.html; 6. BSD100: https://doi.org/10.1109/ICCV.2001.937655; 7. Manga109: https://doi.org/10.1007/s11042-016-4020-z; 8. M3FD: https://openaccess.thecvf.com/content/CVPR2022/html/Liu_Target-Aware_Dual_Adversarial_Learning_and_a_Multi-Scenario_Multi-Modality_Benchmark_To_CVPR_2022_paper.html; 9.Iray infrared super-resolution dataset: http://openai.raytrontek.com/apply/Sea_shipping.html/.
